# Evaluation of biofilm formation in the homozygous and heterozygous strains of vaginal *Candida albicans* isolates

**DOI:** 10.18502/cmm.5.2.1160

**Published:** 2019-06

**Authors:** Keyvan Pakshir, Sahar Sheykhi, Kamiar Zomorodian, Hasti Nouraei, Zahra Zare Shahrabadi

**Affiliations:** 1 Department of Parasitology and Mycology, Basic Sciences in Infectious Diseases Research Center, School of Medicine, Shiraz University of Medical Sciences, Shiraz, Iran; 2 Department of Parasitology and Mycology, School of Medicine, Shiraz University of Medical Sciences, Shiraz, Iran; 3 Department of Parasitology and Mycology, School of Medicine, Tehran University of Medical Sciences, Tehran, Iran

**Keywords:** Biofilm, Candida albicans, Heterozygous, Homozygous, Virulence factor

## Abstract

**Background and Purpose:**

*Candida albicans* is one of the most opportunistic yeasts around the world. This species has two heterozygous and homozygous strains at hyphal wall protein 1 (*hwp1*) gene locus. A simple method for the discrimination of these two strains is the amplification of *HWP1* gene. Regarding this, the aim of this study was to discriminate *C. albicans* heterozygous and homozygous strains via the amplification of *hwp1* gene and evaluation of biofilm formation between the strains.

**Materials and Methods:**

A total of 60 homozygous (n=30) and heterozygous (n=30) strains were discriminated among 126 *C. albicans* vaginal isolates by the amplification of *HWP1* gene, using specific primers. The evaluation of biofilm formation was accomplished using the visual method.

**Results:**

According to the results, the homozygous and heterozygous strains produced one and two DNA fragments, respectively. The frequency of homozygous strains among the *C. albicans* vaginal isolates was 76.2%. Biofilm formation activity in the heterozygous strains was more than that in the homozygous strains. However, statistical analysis showed no significant difference between the strains in terms of biofilm formation.

**Conclusion:**

As the findings indicated, the frequency of the heterozygous strains in *C. albicans* was lower than that of the homozygous strains. Both of the strains could form biofilm in the different ranges of severity. High activity of biofilm formation in heterozygous strains may set the ground for its pathogenicity.

## Introduction

*Candida albicans *is the most pathogenic *Candida *species causing candidiasis in the world. Hyphal wall protein 1 (*HWP1*) is a gene causing virulence in systemic candidiasis [1]. The *HWP1 *also encodes an adhesion receptor which plays a role in adhesion and biofilm formation by cross-linking to the glucans of *C. albicans* cell wall [2]. The genetic characteristics of *C. albicans* as a diploid fungal pathogen has been studied by many researchers [3, 4]. 

*Candida albicans* has two homozygous and heterozygous strains at the *HWP1 *locus. Accordingly, one of the methods for the identification of these strains is the amplification of this gene [5]. In this regard, during the amplification of this locus, the production of one DNA fragment at 941 base pair (bp) reveals homozygous strains, while the production of two fragments at 941 and 839 bp is indicative of the heterozygous strains of *C. albicans* [5-7].

*Candida albicans* has different virulence factors. Secretion of exoenzymes and biofilm production have an important role in the virulence of *C. albicans* [8]. Biofilm formation is thought to be one of the *Candida’s *most important growth adaptations and could serve as a reservoir for disseminated infections [9]. 

*Candida* vaginitis is one of the most common forms of candidiasis in women. In general, 85-90% of the vaginal isolates obtained from patients with *Candida* vaginitis is *C. albicans *[5, 10]. *Candida* vaginitis is the result of the superficial penetration of *Candida* species into the mucosal lining of the vagina and induction of an inflammatory response. The degree of irritation and severity in symptoms is typically different in patients [10]. Adhesion to the surface of the mucosa is the first step for *Candida *invasion. 

A combination of homozygous and heterozygous strains of *C. albicans* almost exists in the vaginal area. The identification of the differences in the severity of adhesion between these strains as a risk factor could be responsible for the severity of symptoms among the patients. With this background in mind, the aim of the current study was to evaluate the prevalence of homozygous and heterozygous strains in vaginal *C. albicans* isolates by the amplification of *HWP1* gene and implementation of a comparative analysis of biofilm as a virulence factor among the isolates. 

## Materials and Methods

The research project was approved by the Ethics Committee of Departmental Review Board (Ethical code: IR.SUMS.REC.1397.380) of Shiraz University of Medical Sciences, Shiraz, Iran.

***Candida albicans isolates***

A total of 30 homozygous and 30 heterozygous strains of vaginal *C. albicans* isolates were estimated for the evaluation of biofilm formation. Given the lack of any distinct data about the frequency of homozygote and heterozygote in *C. albicans*, a total of 126 stock samples of vaginal *C. albicans* species previously isolated from patients suffering vaginal candidiasis were examined in the current study. 

All the isolates had been previously identified as *C. albicans* through conventional (e.g., germ tube test, colony color on chromogenic media, and chlamydoconidia test) and molecular (e.g., polymerase chain reaction-restriction fragment length polymorphism using restriction enzymes) methods and kept at -20℃ as stocks. The isolates were subcultured on Sabouraud dextrose agar (Merck, Germany) before being used.

***Molecular method for discrimination***

Homozygous and heterozygous strains were discriminated by the amplification of *HWP1* gene.

***DNA isolation and polymerase chain reaction***

Genomic DNA was extracted by the boiling method as described by Makimura et al. [11].

To this end, some yeast colonies were added to a lysis buffer containing 30 mM ethylenediaminetetraacetic acid, 0.5% sodium dodecylsulphate, and 100 mM Tris-HCl, and then boiled for 15 min. In the next stage, potassium acetate solution (2.5 M) was added and held on ice for 1 h, followed by centrifugation at 16,128 g for 5 min. Afterward, the DNA in the supernatant was precipitated with isopropanol, washed twice with ethanol, and dried in the air. 

Finally, 50 𝜇l of distilled water was added prior to use for PCR. The amplification of* HWP1 *gene was accomplished using two primers (i.e., CR-f 5'-GCTACCACTTCAGAATCATCATC-3' and CR-r 5'-GCACCTTCAGTCGTAGAGACG-3') and Ampliqon master mix kit (Amplicon, Denmark). The PCR reaction conditions for 35 cycles included initial denaturation for 5 min at 94℃, denaturation step for 30 sec at 94℃, annealing for 45 sec at 62℃, and extension for 45 sec at 72℃, followed by a final extension for 7 min at 72℃. 

The PCR products were separated by gel electrophoresis in a 1.2% (wt/vol) agarose and visualized by staining with ethidium bromide (0.5 𝜇g/ml). In this regard, the production of one DNA fragment at 941 base pare (bp) reveals homozygous strains, while the formation of two fragments at 941 and 839 bp is indicative of the heterozygous strains of *C. albicans*.

***Biofilm formation detection***

Biofilm formation was assessed by a visual detection method [12]. To this end, a loopful of the colonies was added to a 10-mL Falcon tube, containing Sabouraud dextrose broth supplemented with glucose (at a final concentration of 8%), and then incubated at 35℃ for 48 h.

The broth in the tubes was gently aspirated, and the tubes were washed with distilled water twice, and then stained with safranin 1%, which was decanted after 10 min. In the next step, the tubes were examined for the presence of visible adherent films at the bottom and wall of the tubes. The test was conducted in duplicate, and the results were expressed as negative (–), weak (+), moderate (++), and strong (+++). In addition, *Staphylococcus* *epidermidis (*PTCC 1435) and *C. albicans* (ATCC10261) were used as positive controls.

***Statistical analysis***

The data were analyzed using the Fisher’s exact test. A p-value less than 0.05 was considered statistically significant.

## Results and discussion

**Figure 1:**
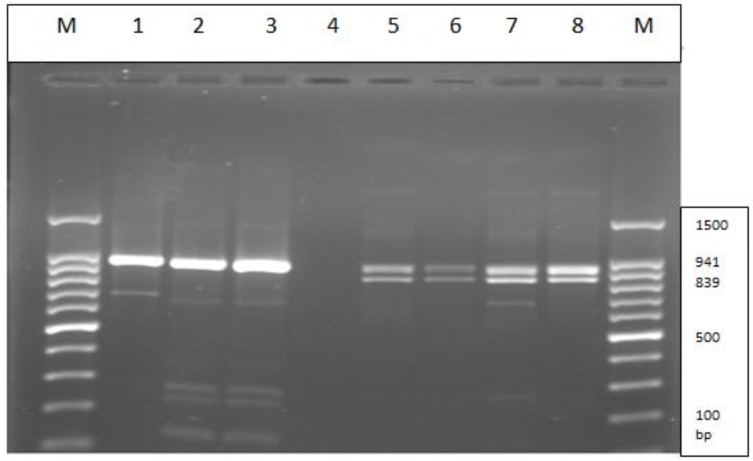
Amplification of *HWP1 *gene producing one fragment (941 bp) for homozygous strain (lanes 2-4) and two fragments (941 and 839 bp) for heterozygous strains (lanes 6-9); molecular size markers (lanes 1, 10)

**Figure 2:**
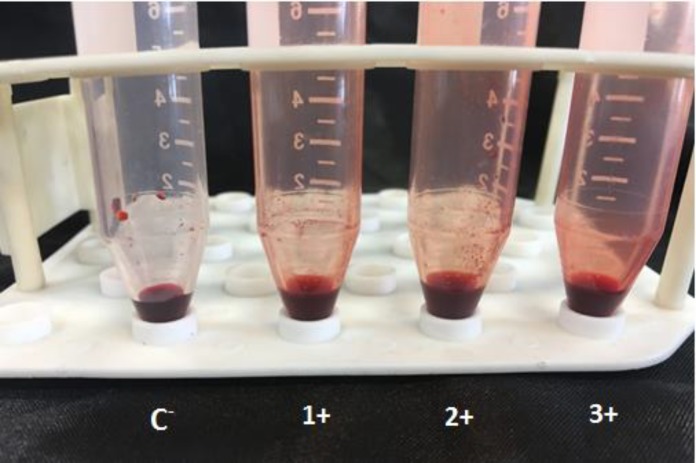
Biofilm formation test [From left to right: Negative control (c), the second (grade 1^+^), the third (grade 2^+^), and the fourth (grade 3^+^)]

According to the results, *HWP1* gene was perfectly amplified in all 126 isolates of *C. albicans. *In addition, 30 homozygous strains and 30 heterozygous strains were discriminated according to the number of DNA fragments (Figure 1). The homozygous and heterozygous strains had the frequencies of 76.2% and 23.8% among the examined *C. albicans* species, respectively. Biofilm formation was detected in 93% of the homozygous strains, where 30% of the strains expressed 3+ activities (Figure 2). Furthermore, 2 homozygous strains were biofilm-negative, and 9 strains had a strong biofilm formation (3+). 

In addition, with regard to the heterozygous strains, all of the strains had biofilm formation, and 50% of the strains presented 3+ activities (Figure 2). More details are available in Table 1. Despite the variety in the results obtained for the two strains, the statistical analysis of biofilm formation demonstrated no significant difference between the two strains (*P*=0.21). 

*Candida albicans* is one of the most common causes of *Candida* infections. The other two yeast species that are closely related to *C. albicans* are* Candida dubliniensis *and* Candida africana, *named as *C. albicans* species complex [5, 13]. One of the best methods for the discrimination of these species is the production of DNA fragments in different sizes through the amplification of *HWP1* gene [14, 15]. The amplification of *HWP1 *gene locus could also facilitate the discrimination of homozygous and heterozygous strains of *C. albicans* by producing one or two DNA fragment size after the emergence of the bands in agarose gel [6].

**Table 1:** Comparison of virulence factors and biofilm formation between the heterozygous and homozygous strains of vaginal *Candida albicans*

**Strains**	**Virulence factors**				**Total**
	**Biofilm formation**				
	**0 -**	**1+**	**2+**	**3+**	
Homozygous	2	10	9	9	30
Heterozygous	0	6	9	15	30

There are many studies regarding the role of *HWP1* gene in *Candida* pathogenesis as a risk factor. Tsuchimori et al. [16] investigated the role of this gene as a virulence factor and reported a reduction in virulence during exposing a homozygous *HWP1 *null mutant of *C. albicans* in infected mice. Furthermore, Hosseini et al. [17] highlighted the importance of *HWP1* gene in coding a cell surface protein, suggesting that biofilm formation is responsible for adherence. Orsi et al. [18] also stated that* HWP1* affects the pathogenic morphological structure of the homozygous and heterozygous genotypes of *C. albicans* and plays a role in yeast adhesion to the epithelial cells.

*Candida* vaginitis, as one of the most common *Candida* infections in women, is caused by many species of *Candida,* especially *C. albicans*. Secretion of extracellular hydrolytic enzymes, biofilm formation, phenotypic switching, adherence to host tissue, and many other factors have been listed as virulence factors. In addition, the aforementioned factors are reported to be involved in the pathogenicity of *Candida* species [19, 20]. Aspartyl proteinase contributes to tissue damage during vaginal candidiasis infection, while the other enzymes induce different tissue damages [21]. However, adherence to the cell surfaces and biofilm production are the first steps for tissue invasion, followed by inflammation as a clinical manifestation. 

To the best of our knowledge, there are no studies investigating exoenzymes as virulence factors in these strains. The results of the current study indicated that the homozygous strains (75%) had a much higher frequency in the vaginal samples than the heterozygous strains. However, both strains were responsible for vaginal candidiasis. Any differences in virulence factors between these two strains could be responsible for the severity of symptoms in patients with vaginal candidiasis. 

Our results revealed that both strains had the ability to produce biofilms in a different range of severity. The ability to form biofilm is an important factor in the pathogenesis of* C. albicans*. Based on our data, biofilm activity was lower in the homozygous strains than in the other strain. In this regard, more than 50% of the heterozygous strains could form biofilms at a high level, while only 6.7% of the homozygous strains were biofilm-negative. The statistical analysis showed no correlation between the two strains in terms of biofilm formation. However, it is hypothesized that the enhancement of the sample size may convert the results into statistically significant ones.

## Conclusion

Based on the findings, the homozygous strains of *C. albicans* species isolated from vaginal candidiasis had a higher frequency as compared to the heterozygous strains. The comparative analysis of biofilm formation demonstrated that most of the strains could produce biofilm in different ranges of adherence. The severity of symptoms in vaginal candidiasis patients could be related to the types of strains. Nonetheless, in this sample size, our data analysis demonstrated no significant difference between the two strains in terms of the severity of biofilm formation. 
